# Analysis of the volatile organic compounds from leaves, flower spikes, and nectar of Australian grown *Agastache rugosa*

**DOI:** 10.1186/1472-6882-14-495

**Published:** 2014-12-15

**Authors:** Hanaa Yamani, Nitin Mantri, Paul D Morrison, Edwin Pang

**Affiliations:** School of Applied Sciences, Health Innovations Research Institute, RMIT University, Melbourne, 3000 Victoria Australia

**Keywords:** *Agastache rugosa*, Estragole, HS-SPME, Volatile Organic Compounds, Nectar, GC–MS

## Abstract

**Background:**

The foraging choices of honey bees are influenced by many factors, such as floral aroma. The composition of volatile compounds influences the bioactivity of the aromatic plants and honey produced from them. In this study, *Agastache rugosa* was evaluated as part of a project to select the most promising medicinal plant species for production of bioactive honey.

**Methods:**

Headspace solid-phase microextraction HS-SPME /GC-MS was optimized to identify the volatile bioactive compounds in the leaves, flower spikes, and for the first time, the flower nectar of Australian grown *A. rugosa*.

**Results:**

Methyl chavicol (= estragole) was the predominant headspace volatile compound in the flowers with nectar, flower spikes, and leaves, with a total of 97.16%, 96.74% and 94.35%, respectively. Current results indicate that HS–SPME/GC–MS could be a useful tool for screening estragole concentration in herbal products.

**Conclusion:**

Recently, estragole was suspected to be carcinogenic and genotoxic, according to the European Union Committee on Herbal Medicinal Products. Further studies are needed on safe daily intake of *Agastache* as herbal tea or honey, as well as for topical uses.

## Background

*Agastache rugosa*, known as Korean mint, is an ornamental plant of the Lamiaceae family that is native to Korea, Japan, and China. It is widely distributed throughout East Asia and used as a traditional ornamental and medicinal plant. It is also commercially cultivated for flavouring agents and as a source of food spice [1]. Several studies have suggested that *A. rugosa* exhibits a variety of pharmacological and physiological activities, such as antifungal [2], antibacterial [3], anticancer [4] and antiviral [5] properties. It is used as a traditional herbal drug for the treatment of intestinal disorders, anorexia, and vomiting [6]. Furthermore, recent studies have shown that *A. rugosa* exhibits anti-inflammatory and anti-atherogenic properties, due to high levels of tilianin [1, 7].

In fact, the composition of volatile compounds influences the bioactivity of aromatic herbs and the essential oils produced from them. The phytotoxic and antimicrobial activities of the essential oils from the leaves of *A. rugosa* could result from estragole, which is the predominant volatile compound, or estragole in combination with small quantities of terpenoids [6]. Korean *A. rugosa* plants are grouped into five chemo types: Methyl chavicol (= estragole), methyl eugenol, methyl eugenol plus limonene, menthone, and menthone plus pulegone [8] In addition, the foraging choices of honey bees are influenced by many factors, such as floral aroma. Also, foraging bees can distinguish between two volatile oil chemo types of the same plant species [9]. *Agastache* species have been suggested for large-scale cultivation as a source of nectar for honey bees by different publications from many nations [7].

Naturally occurring genotoxic and carcinogenic volatile compounds such as estragole are often present in aromatic plants. Several studies have shown the carcinogenicity of estragole in experimental animals after a few reported doses, and after chronic exposure in bacteria and yeast cells [10]. In addition, the metabolites of estragole, such as 1-hydroxy-estragole; 1-hydroxy-2′, 3′-dihydro- estragole; and 1-acetoxy- estragole have more hepatocarcinogens than estragole [11]. Indeed both *in vitro* and *in vivo* studies have demonstrated the formation of hepatic DNA adducts by those metabolites and have defined the major DNA adducts in hepatic cells [11, 12].

There are considerable differences between volatiles extracted from fresh living material and the essential oils obtained from the same plant. Wilson et al. conducted a study to determine the effectiveness of headspace solid-phase microextraction (SPME) analysis in combination with gas chromatography–mass spectrometry to identify volatile compounds given off by inflorescences and leaves compared with traditional volatile oil extraction methods [7]. SPME is a technique first introduced by Pawliszyn [13]. It is especially valued by the food industry as a less expensive, rapid, and solvent-free technique for analysing the fractionation of volatile compounds in different samples [14]. The volatile compounds present in the essential oils from the leaves and flower spikes of *A. rugosa* have been identified by many studies from different areas of the world. However, few studies have examined the headspace volatile compounds of fresh leaves and flower spikes using Headspace solid-phase microextraction [HS-SPME].

To the best of our knowledge, this research study, for the first time, analysed the volatile compounds present in the flowers with nectar and compared them to the compounds present in flower spikes and leaves of Australian grown *A. rugosa*. HS-SPME /GC-MS was used to optimize the suitable conditions for extracting the volatile compounds and to measure particularly the amount of estragole and other volatile compounds present in Australian *A. rugosa* samples. It was evaluated as part of a project to select the most promising medicinal plant species to produce bioactive honey from.

## Methods

### Samples

*Agastache rugosa*, used in this experiment was verified and vouchered at the Medicinal Plant Herbarium, Southern Cross Plant Science (Southern Cross University, New South Wales, Australia). The voucher number is PHARM-14-0029. Fresh leaves, flower spikes, and flowers with nectar of *A. rugosa* were collected from the Chinese medicinal garden at the Royal Melbourne Institute of Technology (RMIT University, Melbourne, Australia) in the summer of 2012; the temperature range ranged from 22–35°C. To collect flowers with nectar, the inflorescences were covered with mosquito nets for 24 h before collecting the flowers the next morning (at 9:00 am) in order for nectar accumulation. To collect flower spikes without nectar, the spikes were collected at the budding stage before nectar secretion began [15]. The samples were kept on ice during collection and transported to the laboratory, where 0.15 g of the inflorescence or freshly ground leaf material was placed into a 4-ml clear, screw-top vial and sealed with a black polypropylene open-top cap and a PTFE (Polytetrafluoroethylene)/silicone septum (Agilent Technologies, Santa Clara, CA).

### Isolation of volatile compounds

Extraction of the volatiles from the ground leaf material, flower spikes and flowers with nectar was performed by HS-SPME. An 85-μm polyacrylate (PA) fibre was fitted to a manual sampling fibre holder (Supelco, Bellefonte, PA). The fibre was conditioned according to the manufacturer’s instructions before use.

### Extraction by HS-SPME

**Figure 1:**
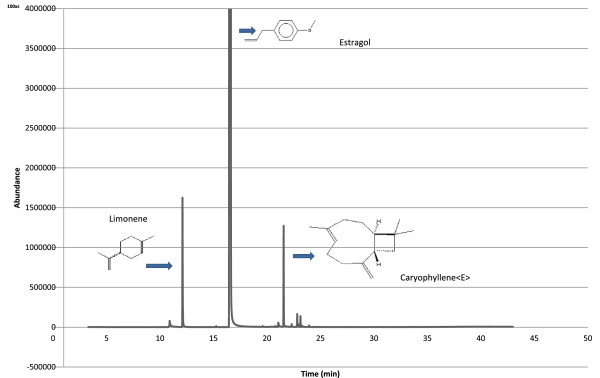
**Chromatographic profile for extraction of the flower nectar using PA fibre in SPME/GC-MS.**

The preconditioned PA fibre was inserted into the headspace of the vial containing the sample, and the vial was placed in a heating block at 40°C for 50 min. The volatiles were desorbed by placing the fibre into the gas chromatography (GC) injection port for 5 min. The equilibrium time profile (solid matrix) was examined using the method of Da Porto & Decorti, with some modification [16]. The vials were placed in the heating block at 40°C instead of 30°C in order to extract all the compounds that might be present under hot temperatures on summer temperatures in Melbourne. Moreover, heating at 40°C resulted in an increased amount of volatile compounds on the fibre and a higher number of resulting peaks compared to heating at 30°C. Figure 1 demonstrates a chromatographic profile for extraction of the flowers with nectar using PA fibre in HS-SPME after optimisation.

### Gas chromatography–mass spectrometry

Identification of the volatile compounds was performed using an Agilent 5973 MSD fitted with a DB-5 MS (5%-phenyl)-methylpolysiloxane fused silica column (Agilent) (30 m x 250 μm i.e., film thickness 0.25 μm). GC–MS data was obtained under the following conditions: carrier gas helium (He 99.99%); flow rate 1.5 ml/min; spilt ratio 50:1. The initial oven temperature was 40°C for 3 min, after which it was raised from 40°C to 250°C at 6°C/min, where it was held for 5 min. The injection port, transfer line, and source temperatures were 250°C, 280°C, and 230°C, respectively. The mass scan range was 41–415 m/z as per Adams: Essential Oil Components by Quadrupole GC/MS. Data acquisition and processing were performed using MSD ChemStation (E02.00.493) (Agilent Technologies, Mulgrave, Australia).

### Identification of the volatile compounds

Qualitative identification was performed using GC–MS reference libraries (Adams [17], Wiley 7th, and NIST 2.0) using a 80% similarity match cut off value. Concentrations of the studied compounds were calculated from the peak areas in the total ion chromatograms. The relative abundance was obtained from electronic integration measurements and the results are the average of three replicates.

### Retention indices

Kovats standard retention indices were determined from the retention times of a series of n-alkane mixture analysed under identical conditions [17].

## Results and discussion

### Volatile composition

**Figure 2:**
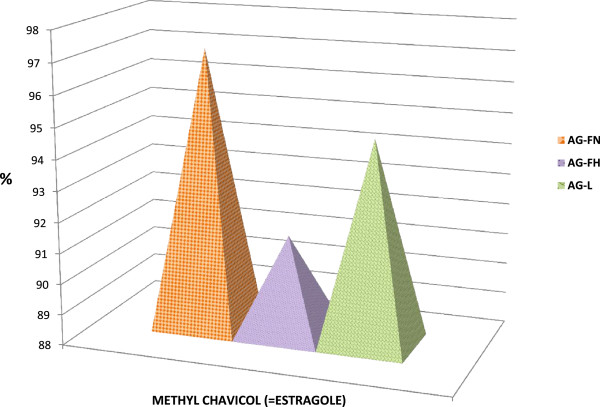
**Comparison between estragole composition percentage: present in flower spikes, flower nectar and leaves of** ***A. rugosa*** **.**

In order to determine the amount of estragole and other volatile compounds presents in the Australian-grown *A. rugosa* flowers with nectar, flowers spikes, and leaves, the samples were subjected to HS–SPME/GC–MS analysis. Seventeen volatile compounds were identified where the range of estragole was 94–97% (Table 1). Previously, many studies indicated that estragole is the most abundant of the *A. rugosa* essential oil volatile compounds ranging from 56-94% [8]. In full agreement with those previous studies, estragole was the predominant headspace volatile compound in all samples tested by HS–SPME in this study. Estragole occurred in slightly different concentrations in the flowers with nectar (97.16%), flower spikes (96.74%) and leaves (94.35%) as shown in Figure 2.

**Table 1:** **Volatiles compounds extracted from flower spikes, flower with nectar and leaves of** ***A. rugosa*** **using HS-SPME/GC-MS**

NO.	Compound (Adams KI)	LRI	PtR	Flower spikes	Flowers + Nectar	Leaves
1	artemisia triene	930	9.28	0.03		0.3
2	bicyclogermacrene	1500	23.17	0.31	0.12	0.38
3	bisabolene, b-	1509	23.24	0.01	0.04	
4	bisabolol, a-	1683	26.57	0.02	0.06	
5	caryophyllene < ( E )->	1419	21.38	1.45	0.84	1.09
6	germacrene D	1480	22.65	0.28	0.13	0.16
7	gurjunene, <gamma>	1477	22.58		0.05	
8	humulene, <alpha->	1455	22.13	0.05	0.03	0.03
9	Limonen	1031	12.1	1.35	1.74	0.84
10	methyl chavicol (= estragole)	1195	16.33	96.74	97.16	94.35
11	methyl eugenol	1401	21.01	0.22	0.11	0.16
12	muurolene < gamma->	1480	22.65	0.03		0.03
13	octanone, <3->	986	10.87			0.12
14	octen 3 ol <1->	979	10.67	0.02		0.8
15	octen-3-yl acetate,< 1->	1110	14.2			0.11
16	hexanol < n->	871	7.77			0.19
17	hexenal < 2E->	855	7.41			1.52

Results are the average of three replicates.

LRI – Linear retention index.

PtR - Predicted retention time.

**Figure 3:**
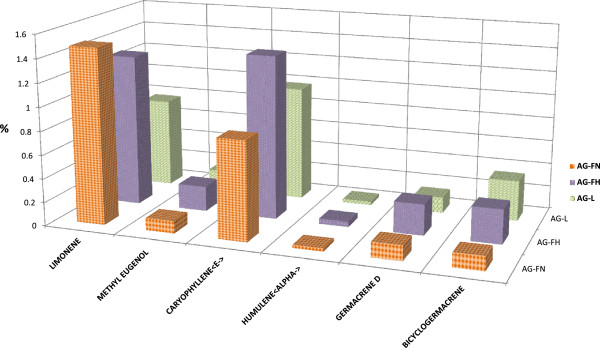
**Comparison between other volatiles compounds percentage: present in flower spikes, flower nectar and leaves of** ***A. rugosa*** **.**

Sixteen other trace volatile compounds were detected in amounts less than 1% in all of the samples analysed (Figure 3). Six volatile bioactive compounds were present in all of the samples, but at different concentrations. Some compounds, such as bicyclogermacrene, were more prevalent in the leaves than in the flower spikes and flowers with nectar, possibly because the compound transferred from the leaves to the other parts of the plant’s inflorescences. However, limonene was more prevalent in the flowers with nectar than in the flower spikes and leaves (1.74%, 1.35%, and 0.84%, respectively), and β-caryophyllene was more prevalent in the flower spikes than in the leaves and flowers with nectar (1.45%, 1.09%, and 0.84%, respectively). On the other hand, some compounds were present only in the leaves, flower spikes, or flowers with nectar. For example, hexanol < n- > and hexenal < 2E- > were present in the leaves only. This is not surprising given that these two compounds are known as the green odour compounds (Table 1).

### Comparison of volatile compositions in different locations

The concentrations of estragole compared with the other volatile compounds differed according to the location, particular plant organ, season, and differences among individuals [18, 19]. Our results were similar to the results of Charles et al. who identified estragole, limonene, and β-caryophyllene as the major volatile compounds in *A. rugosa* grown in the USA [20]. Ahn and Yang analysed the essential oils of Korean *A. rugosa* by GC–MS and found that estragole was the major volatile compound, at more than 90%, followed by limonene (1.4–4.7%) and eugenol (1.4–1.9%) [21]. However, Jun et al. also analysed the essential oils of Korean *A. rugosa* using GC–MS, and they found that limonene was the major compound, at 47%, and that it contained high amounts of caryophyllene (17%) and germacrene-D (14%) [1]. They also found some other minor compounds, such as chavicol, bourbonene, and eugenol, for a total of 22%.

Generally, the volatile compounds produced from inflorescences are two to six times more volatile per gram than those found in the leaves. Our results were similar to those reported by (Vlietinck & Kubelka 2004) [22] (1996) for the essential oils found in the leaves and flowers of *A. rugosa* in Vietnam [23]. They also found that the essential oil in the leaves and flowers was characterised by a high amount of estragole; however, they reported greater amounts in the leaves than in the flowers, whereas in the current study, less estragole was extracted from the leaves by HS–SPME than from the flower spikes and flower with nectar.

### HS–SPME

In this study, HS–SPME conditions were optimized to extract the volatile bioactive compounds instead of using traditional essential oil extraction and solvent extraction methods. Zielińska et al. extracted the volatiles of Korean *A. rugosa* leaves using the same HS–SPME/GC–MS technique, but they used a fibre coated with 50/30-μm divinylbenzene-carboxen-polydimethylsiloxane for the extraction process [24]. Despite using different fibres and different conditions, both studies produced similar results. In their study, estragole made up 95% of the total volatile compounds extracted from the leaves, and ten other volatile compounds were extracted from the leaves, eight of which were the same as the volatiles extracted by the current study. These results indicate that HS–SPME/GC–MS could be a useful tool for screening estragole concentration in herbal products.

### Estragole toxicity

There is only one study using humans, which reported the amount of 1-hydroxyl-estragole in the urine after oral administration of estragole to two volunteers. After oral administration of 100 μg/day for six months, the urinary extraction of 1-hydroxyl-estragole amounted to 0.2% and 0.4% of the total administered dose, corresponding to the average exposure levels of humans [25]. The profile of metabolism and metabolic activation has been clearly established by several studies, showing that covalent binding is dose-dependent. In particular, a study using mice showed that the carcinogenicity of estragole is probably minimal in the dose range of 1–10 mg/kg body weight, which is approximately 100–1000 times the expected exposure to estragole from short-term use in adults at dosage recommendations [22].

## Conclusion

To the best of our knowledge, this study is the first to determine the volatile compounds in the flower nectar from Australian-cultivated *A. rugosa* and compare these with the amounts in the leaves, flower spikes and flowers with nectar. HS–SPME/ GC-MS conditions were optimized to extract the volatile bioactive compounds instead of using traditional essential oil extraction and solvent extraction methods. In all of the samples tested, estragole was present at a high concentration, ranging 94–97%. In 2000, the Committee of Experts on Flavouring Substances (CEFS) evaluated estragole and recommended a limit of 0.05 mg/kg (detection limit). In addition, in 2004, the European Agency for the Evaluation of Medicinal Products (EMEA) suggested minimal exposure of estragole to sensitive groups such as children, pregnant and breastfeeding women. Although estragole does not pose a remarkable cancer risk in short-term use by dosage recommendations, more in vivo and in vitro studies are needed to determine the risk associated with long-term exposure to estragole with a wide range of doses. Further studies are also needed to investigate external and topical uses, as well as daily intake when used as an herbal tea or honey.

## Abbreviations

A. rugosa: *Agastache rugosa*
HS-SPME: Headspace solid-phase microextraction
GC–MS: Gas chromatography–mass spectrometry
PTFE: Polytetrafluoroethylene.
